# Thoracic Skeletal Muscle Attenuation Predicts Sex-Specific Mortality After Coronary Artery Bypass Grafting—Sex-Specific Insights into Risk Stratification and Frailty Assessment

**DOI:** 10.3390/medsci14050569

**Published:** 2026-09-14

**Authors:** Philipp Krombholz-Reindl, Andreas Winkler, Christian Dinges, Klaus Linni, Manuela Pilz, Stephanie Rassam, Matthias Hammerer, Nikolaus Clodi, Roman Gottardi, Stefan Hecht, Johannes Holfeld, Elke Boxhammer, Andreas Voetsch

**Affiliations:** 1 Department of Vascular and Endovascular Surgery, Paracelsus Private Medical University Salzburg, 5020 Salzburg, Austria; 2 Department of Cardiac Surgery, Paracelsus Private Medical University Salzburg, 5020 Salzburg, Austria; 3 Department of Cardiology, Paracelsus Private Medical University Salzburg, 5020 Salzburg, Austria; 4 Department of Cardiac Thoracic and Vascular Surgery, Westpfalz-Klinikum Kaiserslautern, 67655 Kaiserslautern, Germany; 5 Department of Radiology, Paracelsus Private Medical University Salzburg, 5020 Salzburg, Austria; 6 Department of Cardiac Surgery, Karl-Landsteiner University St. Pölten, 3100 St. Pölten, Austria

**Keywords:** coronary artery bypass grafting, Hounsfield Unit Average Calculation, sex-specific, frailty

## Abstract

**Background:** Frailty and impaired muscle quality are strong determinants of adverse outcomes after coronary artery bypass grafting (CABG), yet conventional risk scores overlook this biological dimension. The Hounsfield Unit Average Calculation (HUAC) obtained from routine preoperative computed tomography (CT) scans quantifies skeletal muscle attenuation, reflecting intramuscular fat infiltration and metabolic reserve. This study evaluated the prognostic value of HUAC measured at two thoracic levels and explored sex-specific differences in its predictive performance. **Methods:** In this retrospective single-center study, 479 patients (382 men, 97 women) undergoing isolated CABG with available preoperative CT scans were analyzed. Mean HUAC was measured bilaterally at the fourth (TH4) and twelfth (TH12) thoracic vertebral levels. Sex-specific HUAC quartiles were generated, with the lower quartile defining low thoracic muscle attenuation, representing a radiographic phenotype of impaired muscle quality. Associations with 1-, 3-, and 5-year mortality were assessed by Kaplan–Meier analysis, receiver-operating-characteristic (ROC) curves, and sex-stratified Cox regression models incorporating inverse probability weighting (IPW). Incremental prognostic value beyond EuroSCORE II was assessed by comparing Harrell’s C-statistics of Cox models with and without low HUAC. **Results:** Patients with low HUAC showed reduced survival at all time points. HUAC measured at TH4 demonstrated higher discriminative ability compared with TH12. In women, TH4 yielded area under the curve (AUC) of 0.773 and 0.807 for 1- and 3-year survival, respectively, compared with 0.670 and 0.640 in men. In sex-specific Cox analyses, women in the lower HUAC-TH4 quartile had markedly increased mortality risk (unweighted HRs 6.6, 9.5, and 6.4 for 1-, 3-, and 5-year follow-up; all *p* ≤ 0.03), which remained significant after IPW adjustment. In men, the association was weaker and lost significance after weighting. Formal interaction testing demonstrated significant sex × low-HUAC interactions at TH4 for 3- and 5-year mortality (*p* interaction = 0.046 and 0.045, respectively), whereas no significant interaction was observed at TH12. Addition of low TH4 HUAC to EuroSCORE II improved model discrimination in the overall cohort, with C-statistics increasing from 0.665 to 0.711 at 3 years and from 0.658 to 0.690 at 5 years (both likelihood-ratio *p* < 0.001); the largest incremental gains were observed in women (ΔC = +0.115 and +0.081, respectively). **Conclusions:** Low thoracic muscle attenuation, particularly HUAC at TH4, independently predicts long-term mortality after CABG. Formal interaction testing supports an exploratory sex-dependent prognostic association at TH4, with a stronger association of low muscle attenuation with mortality in women than in men. HUAC represents a simple, objective biomarker of physiological reserve that can be derived from existing preoperative CT scans and may enhance individualized risk stratification in contemporary cardiac surgery.

## 1. Introduction

Coronary artery bypass grafting (CABG) remains a physiological stress test that challenges even modern medicine’s limits of resilience. Despite major advances in surgical technique, anesthesia, and perioperative care, morbidity and mortality after CABG remain substantial, particularly among elderly patients [1,2]. As life expectancy rises, the typical CABG candidate becomes increasingly frail, reflecting not only chronological aging but also a decline in physiological reserve [3]. Yet conventional risk scores such as the EuroSCORE II and STS score still rely largely on demographic and cardiac variables, overlooking this crucial biological dimension of patient resilience [4].

Importantly, comprehensive preoperative risk assessment in patients undergoing CABG extends beyond conventional surgical risk scores and requires consideration of the individual cardiovascular and vascular risk profile. Concomitant vascular disease may necessitate dedicated preoperative assessment and individualized management strategies, including staged interventions in selected patients with relevant carotid artery disease [5]. Nevertheless, even comprehensive clinical and vascular risk assessment may not fully capture biological vulnerability and physiological reserve.

At the core of this reserve lies skeletal muscle, the body’s largest metabolic organ and a crucial determinant of surgical recovery. Beyond locomotion, skeletal muscle governs glucose metabolism, protein turnover, and inflammatory balance [6]. Sarcopenia, the loss of skeletal muscle mass and quality, represents the biological substrate of frailty and has been consistently associated with increased postoperative complications, prolonged hospitalization, and mortality across surgical disciplines [7]. In cardiac surgery, sarcopenia amplifies susceptibility to infection, impairs ventilatory mechanics, and blunts the physiological response to stress [8]. Nevertheless, it is rarely assessed in daily practice because traditional methods such as grip strength, gait speed, or bioimpedance are cumbersome, time consuming and poorly suited for preoperative evaluation [9].

Modern imaging offers a unique opportunity to assess skeletal muscle quality objectively and effortlessly. When clinically available as part of preoperative assessment, computed tomography (CT) inherently contains quantitative information about muscle composition. The Hounsfield Unit Average Calculation (HUAC) quantifies mean muscle attenuation in Hounsfield units (HU), reflecting the degree of intramuscular fat infiltration and metabolic decline [10]. A lower HUAC value indicates poorer muscle quality—a radiological signature of systemic frailty. Unlike surrogate measures of muscle mass, HUAC captures the quality rather than just the quantity of muscle tissue, integrating both structural and metabolic aspects of the patient’s condition [11,12].

While prior research has predominantly focused on abdominal muscles, particularly the psoas and paraspinal regions at the lumbar level [12,13], such measurements are rarely available in preoperative cardiac imaging. By contrast, the thoracic musculature, visualized at the fourth (TH4) and twelfth (TH12) thoracic vertebrae, can be assessed on preoperative chest CT scans when available. These regions therefore represent an ideal, underused site for quantifying skeletal muscle attenuation in the cardiac surgical population [14,15]. Thoracic HUAC measurements are reproducible, require no additional imaging, and can be obtained quickly from existing data without additional cost, time, or patient risk.

The physiological plausibility of HUAC as a prognostic marker in CABG is compelling. Reduced muscle density mirrors systemic inflammation, oxidative stress, and hormonal dysregulation which might impair myocardial recovery and wound healing [16]. Moreover, sarcopenic patients frequently present with anemia [17], renal dysfunction [18], and malnutrition [19], forming a constellation of vulnerabilities that may amplify operative risk. Despite this biological rationale, evidence on HUAC-derived muscle attenuation in CABG patients remains scarce, and the relative prognostic importance of different thoracic levels (TH4 vs. TH12) and sex-specific effects has never been clearly defined.

## 2. Aim

The present study therefore sought to determine the prognostic significance of low skeletal muscle attenuation assessed by HUAC measured at TH4 and TH12 in patients undergoing isolated CABG. Specifically, we investigated its association with mid- and long-term mortality and with key postoperative complications. In addition, we explored sex-specific differences in predictive accuracy to test whether HUAC performs equally well in men and women. By integrating muscle quality, an often overlooked dimension of physiological health, into cardiac surgical risk assessment, this study aims to close the gap between anatomical success and biological resilience.

## 3. Material and Methods

**Figure 1:**
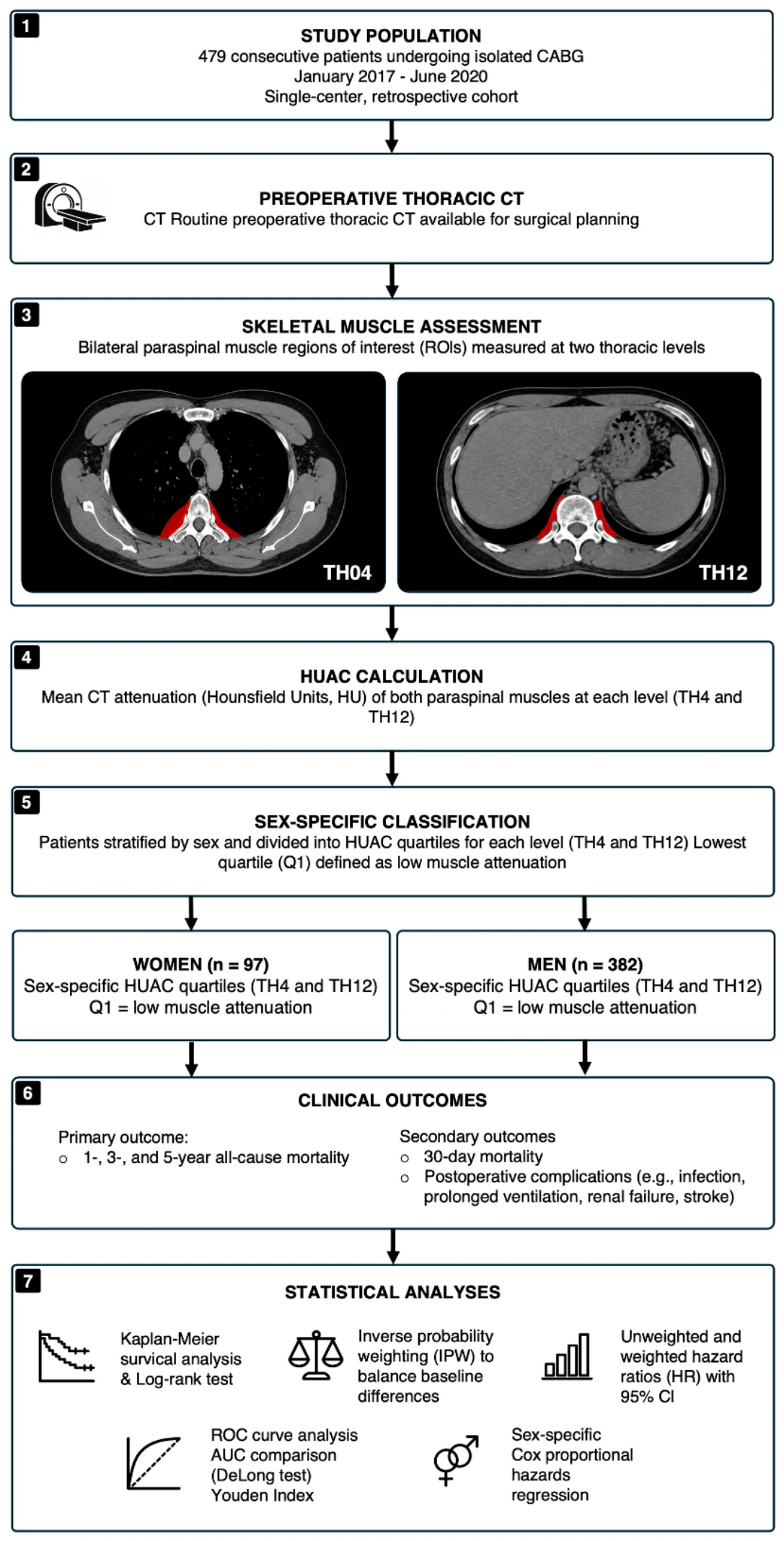
Study design and analytical workflow. Overview of the study population, preoperative thoracic CT assessment, HUAC measurement at TH4 and TH12, sex-specific classification, clinical outcome assessment, and statistical analyses. Abbreviations: CABG = coronary artery bypass grafting; CT = computed tomography; HUAC = Hounsfield Unit Average Calculation; TH = thoracic vertebral level; IPW = inverse probability weighting; ROC = receiver operating characteristic; AUC = area under the curve; HR = hazard ratio; CI = confidence interval.

An overview of the study design, CT-based skeletal muscle assessment, outcome evaluation, and statistical analysis is provided in Figure 1.

### 3.1. Study Population

This retrospective single-center study included 479 consecutive patients between January 2017 and June 2020 who underwent isolated CABG at the Department of Cardiac, Vascular and Endovascular Surgery at Paracelsus Medical University, Salzburg, Austria. All patients had complete preoperative CT scans of the thoracic aorta as part of standard surgical planning and were included if image quality allowed reliable quantification of muscle attenuation and area at the thoracic levels of interest.

Patients who underwent concomitant valve procedures, emergency CABG following acute ST-elevation myocardial infarction, cardiogenic shock or who had missing clinical or follow-up data were excluded.

### 3.2. Ethics Declaration

The study was approved by the Ethics Committee of the federal state of Salzburg, Austria (Ethikkommission für das Bundesland Salzburg; reference number: 1168/2020, approval date: 25 November 2020). All analyses were performed in accordance with the Declaration of Helsinki and the International Council for Harmonisation—Good Clinical Practice (ICH-GCP) guidelines. Given the retrospective design and use of anonymized clinical data, the requirement for individual informed consent was waived.

### 3.3. Data Extraction

Clinical, laboratory, and perioperative data were retrieved from the institutional ORBIS electronic medical records system (Agfa Healthcare, Version 08043301.04110DACHL, Bonn, Germany) and from the hospital’s digital archiving platform (Softworx by Andreas Schwab™, Version 1). Extracted variables included patient demographics, cardiovascular risk factors, preoperative laboratory values, echocardiographic parameters, and operative details.

Postoperative events and in-hospital outcomes were cross-checked using operative reports, progress notes, and discharge documentation. Long-term survival data were obtained through linkage with the Austrian national death registry to ensure complete follow-up.

### 3.4. Patient Sample Collection and Image Analysis

Preoperative CT imaging was performed using contrast-enhanced ECG-gated CT angiography of the thoracic aorta as part of routine surgical planning. CT acquisition was performed with patient size-adapted tube voltage (80–120 kVp) and active tube-current modulation. A total of 80 mL of non-ionic iodinated contrast medium (Visipaque 320 mg I/mL; GE Healthcare, Munich, Germany) was administered intravenously as a bolus. For skeletal muscle assessment, axial images reconstructed at a slice thickness of 3 mm were used. All HUAC measurements were performed in the same contrast phase to minimize contrast-related variability in muscle attenuation.

Preoperative contrast-enhanced CT scans of the thorax were analyzed to quantify skeletal muscle density. Axial images at the fourth thoracic vertebra (TH4) and twelfth thoracic vertebra (TH12) levels were selected. Circular regions of interest (ROIs) were manually placed within the paraspinal muscles on both sides, carefully excluding visible fat and bone. Mean attenuation values in Hounsfield Units (HU) were averaged to obtain the Hounsfield Unit Average Calculation (HUAC) for each level.

All measurements were performed independently by two experienced observers blinded to all clinical data and outcomes. Interobserver agreement was verified by intraclass correlation analysis.

### 3.5. Surgical Procedure

The decision to proceed with surgery was made by a specialized multidisciplinary heart team [20] consisting of a cardiac surgeon, cardiologist, and cardiac anesthesiologist in accordance with current guidelines [21,22]. The vast majority of patients underwent isolated CABG via median sternotomy under general anesthesia, while a small subset was operated through a left thoracotomy. Standard cardiopulmonary bypass with moderate hypothermia (32 °C) was used in the majority of cases, employing crystalloid cardioplegia according to surgeon preference. Conduit selection (left internal thoracic artery [LIMA], right internal thoracic artery [RIMA], saphenous vein grafts, or radial artery) and the number of grafts were determined based on coronary anatomy and intraoperative assessment. The surgical strategy followed European Society of Cardiology (ESC) and European Association for Cardio-Thoracic Surgery (EACTS) guidelines [20].

### 3.6. Outcome Definition

The primary endpoint of the study was all-cause mortality at 1-, 3-, and 5-year follow-up. Mortality data were verified using hospital records and national death registries to ensure complete and accurate ascertainment.

Secondary outcomes included short-term postoperative complications and in-hospital events such as 30-day mortality, perioperative myocardial infarction (MI, Type V following the fourth universal definition of myocardial infarction 2018), new-onset atrial fibrillation (AF), acute kidney injury requiring hemodialysis, wound infection, reintubation or prolonged mechanical ventilation for ≥48 h, major neurological events (defined as modified Rankin Scale ≥ 2 at 90 days postoperatively), and length of stay in the intensive care unit (ICU).

### 3.7. Statistical Analysis

Statistical analyses were conducted using SPSS software (Version 25.0, SPSS Inc., Armonk, NY, USA) and R (Version 4.2.3, R Foundation for Statistical Computing, Vienna, Austria).

The Kolmogorov–Smirnov–Lilliefors test was used to evaluate the distribution of variables. Continuous variables with a normal distribution were reported as mean ± standard deviation (SD) and compared using unpaired Student’s *t*-tests. Non-normally distributed variables were presented as median with interquartile range (IQR) and analyzed using the Mann–Whitney U test. Categorical variables were summarized as frequencies or percentages and assessed using the chi-squared test.

To account for established sex-related differences in skeletal muscle composition and attenuation, HUAC values were stratified into sex-specific quartiles. In the absence of externally validated HUAC thresholds for thoracic muscle attenuation in patients undergoing CABG, the lowest sex-specific quartile (Q1) was prespecified as an internally derived marker of low muscle attenuation. This relative threshold was chosen to identify patients at the lower end of the sex-specific HUAC distribution and should be regarded as an exploratory, cohort-derived definition rather than a clinically validated diagnostic cut-off.

Kaplan–Meier survival analysis was performed to compare short- and long-term mortality following CABG between patients with and without low muscle attenuation with log-rank tests and corresponding risk tables provided. This was performed in the overall cohort and according to sex differentiation. To assess the discriminative ability of HUAC at TH4 and TH12 for 1- and 3-year mortality, area under the receiver operating characteristic (AUROC) analyses were performed separately by sex. For each prespecified time point, only patients with known vital status and sufficient follow-up to determine survival at that time point were included; patients censored before the respective time horizon were excluded from that AUROC analysis. Areas under the curve (AUCs), sensitivity, specificity, and the Youden Index (YI) were calculated. Thresholds maximizing the Youden Index were regarded as exploratory, cohort-derived values and not as clinically validated cut-offs.

To address potential confounding arising from differences in baseline characteristics, inverse probability weighting (IPW) was incorporated into sex-specific Cox proportional hazards models.

Separate propensity score models were constructed using logistic regression, with assignment to the lower HUAC quartile as the dependent variable. Covariates included age, preoperative creatinine, hemoglobin, and EuroSCORE II. Covariates for the propensity score models were selected based on their clinical relevance as established markers of operative risk and physiological vulnerability, while maintaining a parsimonious weighting model. EuroSCORE II was included as a multidimensional measure of surgical risk, whereas age, preoperative creatinine, and hemoglobin were included as additional markers of biological, renal, and hematological reserve. Separate propensity score models were constructed within women and men for TH4 and TH12. Covariate balance before and after weighting was assessed using standardized mean differences (SMDs), with an absolute SMD < 0.10 considered indicative of adequate balance.

The distribution of the inverse probability weights was examined for extreme values. Unstabilized weights were used without truncation or winsorization. Mean weights were approximately 2.0 across all sex- and level-specific models, with maximum weights of 22.56 and 10.17 in women and 10.71 and 10.60 in men for TH4 and TH12, respectively. Positivity was assessed by examining the overlap of propensity-score distributions between the low-HUAC and reference groups, with substantial overlap observed in all models and no evidence of deterministic group assignment. Inverse probability weights were calculated as the inverse of the predicted probability of the observed group assignment and applied using the survey package in R. Weighted Cox proportional hazards models were then fitted using the svycoxph function to estimate hazard ratios (HRs) for 1-, 3-, and 5-year mortality. Robust standard errors were used to account for the survey-weighted design. The proportional-hazards assumption was assessed using Schoenfeld residuals, with no evidence of relevant violations. There were no missing data for variables included in the propensity-score or survival models; therefore, no imputation was required for these analyses.

To formally assess whether the association between low HUAC and mortality differed by sex, additional Cox proportional hazards models including sex, low HUAC, and a sex × low-HUAC interaction term were fitted separately for TH4 and TH12 at 1-, 3-, and 5-year follow-up. Women served as the reference category for sex. Interaction effects are reported as HRs with 95% confidence intervals (CIs) and corresponding *p*-values.

Incremental prognostic value beyond established surgical risk assessment was evaluated by comparing Cox models containing EuroSCORE II alone with models additionally including sex-specific low HUAC at TH4 or TH12, separately for 1-, 3-, and 5-year mortality. Analyses were performed in the overall cohort and stratified by sex. Model discrimination was quantified using Harrell’s C-statistic, with ΔC representing the change after addition of low HUAC. Nested models were compared using likelihood-ratio tests.

A two-tailed *p*-value < 0.050 was considered statistically significant for all analyses.

### 3.8. Data Availability and Funding

The data presented in this study are available from the corresponding author upon reasonable request. Public disclosure of the underlying patient data is restricted by the ethics committee approval for ethical and privacy reasons. This academic study did not receive financial support.

## 4. Results

### 4.1. HUAC Measurements and Definition of Low Muscle Attenuation

Pre-operative thoracic CT scans were available for all 479 patients undergoing isolated CABG. Skeletal muscle attenuation was assessed at the fourth (TH4) and twelfth (TH12) thoracic vertebral levels. Mean HUAC values were lower in women than in men at both levels.

Patients in the lowest sex-specific quartile (Q1) were classified as having low muscle attenuation, corresponding to HUAC < −1.5 HU at TH4 and <11.8 HU at TH12 in women, and HUAC < 11.5 HU and <21.8 HU, respectively, in men (Table 1). These cohort-derived thresholds were used for internal risk stratification and do not represent clinically validated diagnostic cut-offs. Functional criteria such as grip strength or gait speed were not available and therefore not included.

### 4.2. Baseline Characteristics

Baseline clinical characteristics are presented in Table 2. Among the 479 patients included in the study, 382 were men and 97 were women. The overall median age was 70.0 [14.0] years, with no significant difference between men (69.0 [15.0] years) and women (70.0 [12.8] years, *p* = 0.068). As expected, women had a markedly smaller body size, with significantly lower height, weight, and body surface area compared with men (all *p* < 0.001). Despite similar body-mass indices, women exhibited a higher predicted operative risk, as indicated by a EuroSCORE II of 2.2 [2.5] versus 1.5 [1.8] in men (*p* < 0.001).

Laboratory findings also showed sex-specific differences. Men had higher median creatinine concentrations (1.0 [0.3] mg/dL vs. 0.8 [0.3] mg/dL; *p* < 0.001) and higher median hemoglobin levels (14.4 [2.0] g/dL vs. 13.4 [1.2] g/dL; *p* < 0.001). The prevalence of common cardiovascular risk factors—including arterial hypertension, diabetes mellitus, dyslipidemia, peripheral arterial occlusive disease, chronic obstructive pulmonary disease, and atrial fibrillation—was comparable between the sexes. However, significant coronary left-main stenosis (≥50%) occurred more often in men than in women (44.5% vs. 29.9%; *p* = 0.009).

Functionally, women presented with more advanced heart failure symptoms, reflected by a higher proportion in NYHA functional class III–IV (*p* = 0.004). Left-ventricular ejection fraction did not differ between men and women.

### 4.3. Intraoperative Data

Intraoperative details are summarized in Table 3. The majority of procedures were performed on-pump (91.6% overall), with no significant difference between men and women (92.4% vs. 88.7%, *p* = 0.233). Only seven operations (1.5%) were performed through a left thoracotomy, a rate comparable between sexes.

The configuration of internal thoracic artery (ITA) grafting varied moderately. A left internal thoracic artery (LIMA) was used in most cases (62.6% overall), and a bilateral ITA (BIMA) strategy in 31.7%. BIMA use was slightly more common among men (33.8%) than women (23.7%), although the difference did not reach statistical significance (*p* = 0.085). Use of a right internal thoracic artery (RIMA) alone was rare (3.1%).

Intra-aortic balloon pump (IABP) support was infrequent and similar between groups (0.8% in men and 1.0% in women, *p* = 0.812). Men and women had nearly identical perfusion and aortic cross-clamp times (median 93 vs. 92 min and 49 vs. 51 min, respectively; both *p* > 0.5).

Blood product transfusion differed noticeably between sexes. Women received significantly more red blood cell (RBC) transfusions than men (0.8 ± 1.6 vs. 0.1 ± 0.5 units; *p* < 0.001), while administration of platelets or fresh frozen plasma was rare and comparable (*p* = 0.982 and *p* = 0.445, respectively). The median number of arterial and venous grafts per patient did not differ significantly between sexes.

### 4.4. Postoperative Outcomes

Postoperative outcomes are summarized in Table 4. The overall 30-day mortality was low (1.9%) and did not differ between men and women (1.8% vs. 2.1%, *p* = 0.882). Major complications such as low cardiac output syndrome requiring ECMO support (1.3% vs. 1.0%), perioperative myocardial infarction (1.3% vs. 3.1%), and acute kidney injury requiring dialysis (1.6% vs. 1.0%) were infrequent and comparable between sexes (all *p* > 0.2).

Sternal wound infections occurred in 3–4% of patients without sex difference, while new-onset atrial fibrillation was the most common event (≈23% in men and 22% in women; *p* = 0.73). Bleeding or tamponade requiring re-exploration was observed only in men (2.1%). Disabling stroke was infrequent in both groups (3.1% vs. 1.3%, *p* = 0.221).

Duration of mechanical ventilation, ICU stay, and total hospitalization were similar between groups. Overall, postoperative morbidity and mortality were low, with no significant sex-related differences.

### 4.5. Mid- and Long-Term Survival

During follow-up, 18 deaths occurred within 1 year, 41 within 3 years, and 64 within 5 years. Among women, 6, 11, and 14 deaths occurred within 1, 3, and 5 years, respectively, compared with 12, 30, and 50 deaths among men.

**Figure 2:**
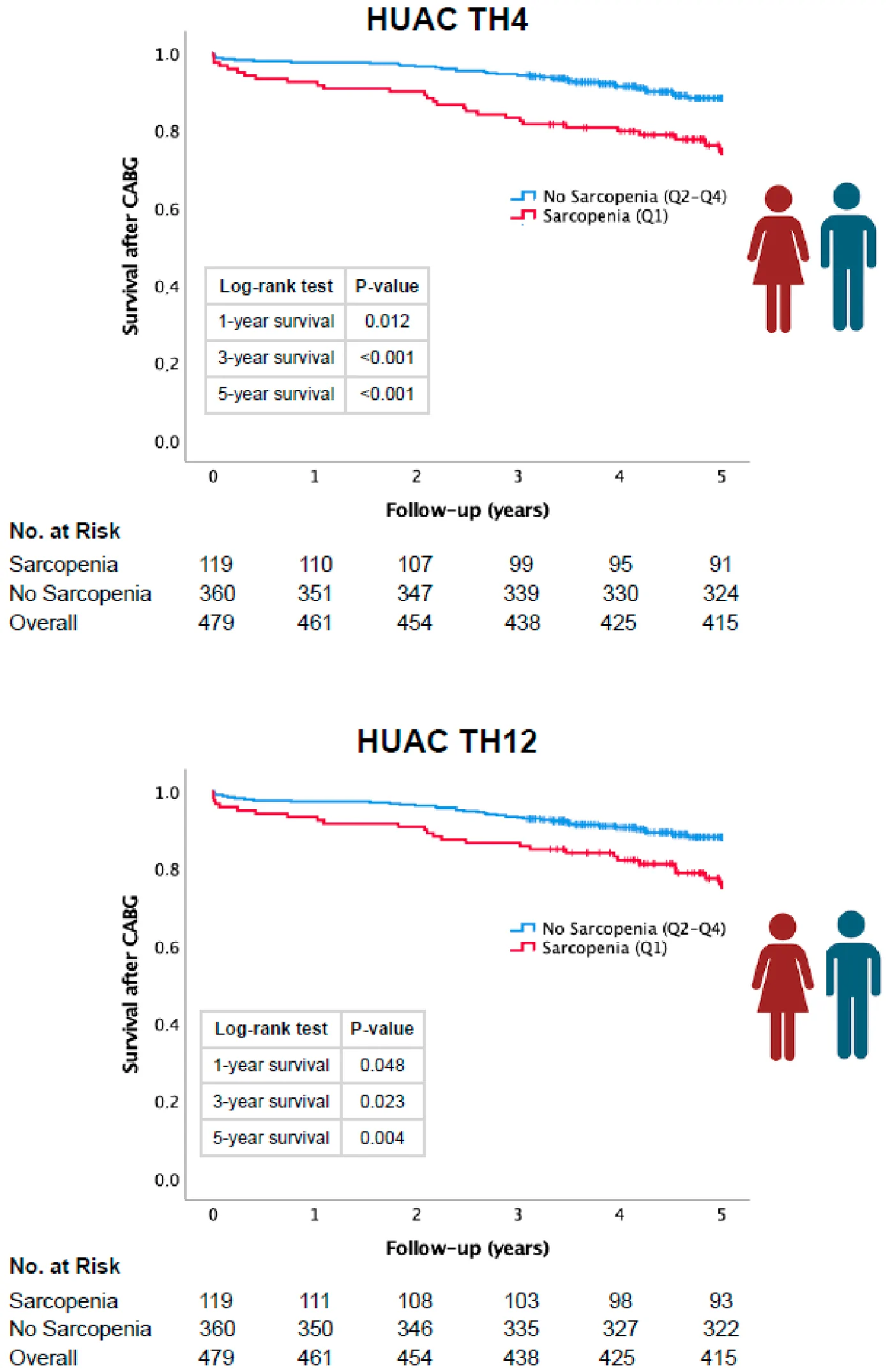
Overall Kaplan–Meier Survival According to HUAC at TH4 and TH12. Kaplan–Meier survival curves for all-cause mortality according to thoracic muscle attenuation (HUAC) measured at TH4 and TH12. Patients in the lower HUAC quartile (sarcopenic group) showed reduced 1-, 3-, and 5-year survival after CABG compared with higher quartiles. Abbreviations: HUAC = Hounsfield Unit Average Calculation; TH4 = fourth thoracic vertebra; TH12 = twelfth thoracic vertebra; CABG = coronary artery bypass grafting.

**Figure 3:**
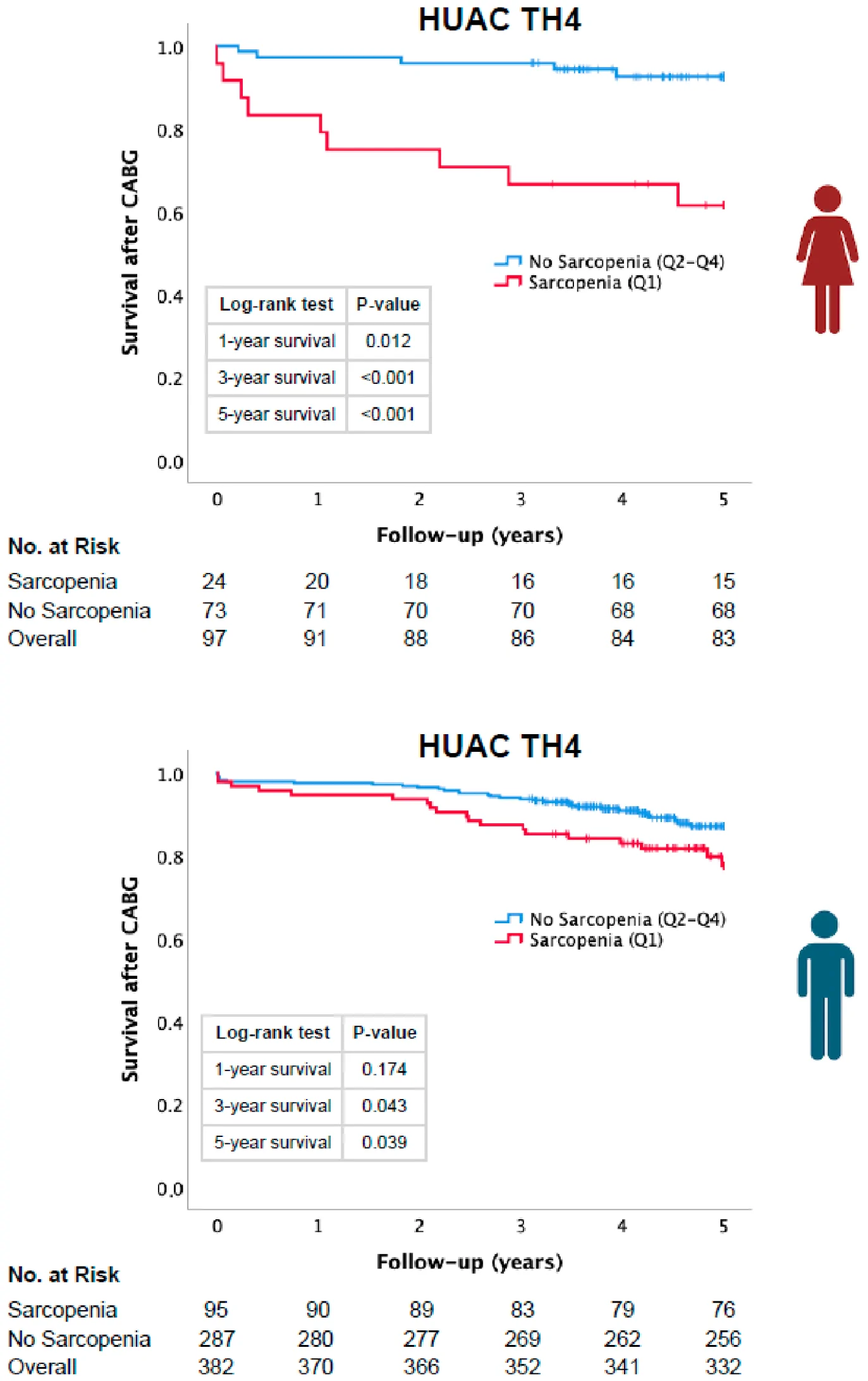
Kaplan–Meier Survival According to HUAC at TH4 Stratified by Sex. Kaplan–Meier survival according to HUAC at TH4, stratified by sex. In both men and women, lower HUAC was associated with increased long-term mortality, with greater separation of the survival curves observed in women. Abbreviations: HUAC = Hounsfield Unit Average Calculation; TH4 = fourth thoracic vertebra.

**Figure 4:**
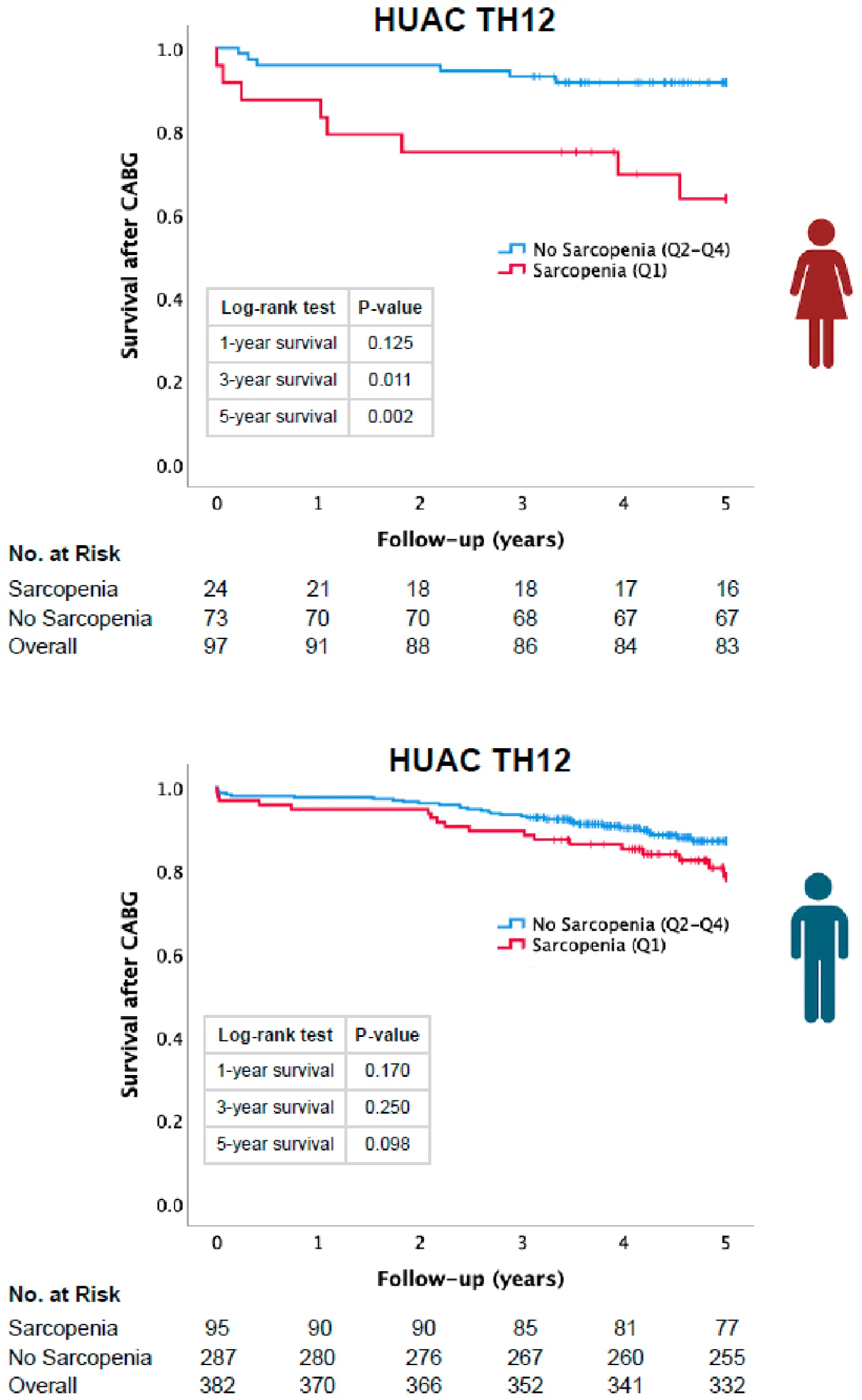
Kaplan–Meier Survival According to HUAC at TH12 Stratified by Sex. Kaplan–Meier survival according to HUAC at TH12, stratified by sex. Low HUAC at TH12 predicted poorer long-term survival, particularly among women. Abbreviations: HUAC = Hounsfield Unit Average Calculation; TH12 = twelfth thoracic vertebra.

Kaplan–Meier analyses demonstrated a clear association between low thoracic muscle density (HUAC) and reduced long-term survival after CABG (Figure 2, Figure 3 and Figure 4).

Patients in the lower HUAC quartile (low-HUAC group) consistently showed poorer survival compared with all others. For HUAC measured at TH4 (Figure 2), survival differences emerged early and widened over time. The log-rank test yielded *p* = 0.048 at 1 year, *p* = 0.023 at 3 years, and *p* = 0.004 at 5 years, confirming a statistically significant separation of curves throughout follow-up.

At the TH12 level, the association was even stronger, with *p* = 0.012 at 1 year and *p* < 0.001 at both 3 and 5 years.

Sex-specific analyses (Figure 3 and Figure 4) revealed the same overall pattern. In men, low HUAC TH4 was linked to progressively reduced survival (*p* = 0.174 at 1 year, *p* = 0.043 at 3 years, *p* = 0.039 at 5 years). For TH12, the effect was weaker and did not reach significance (all *p* > 0.05). In women, greater separation of the survival curves was observed. At TH4, survival differed between the lower and higher HUAC quartiles (*p* = 0.012 at 1 year; *p* < 0.001 at 3 and 5 years), and at TH12 the same trend persisted (*p* = 0.125, *p* = 0.011, and *p* = 0.002, respectively).

### 4.6. ROC Analysis

**Figure 5:**
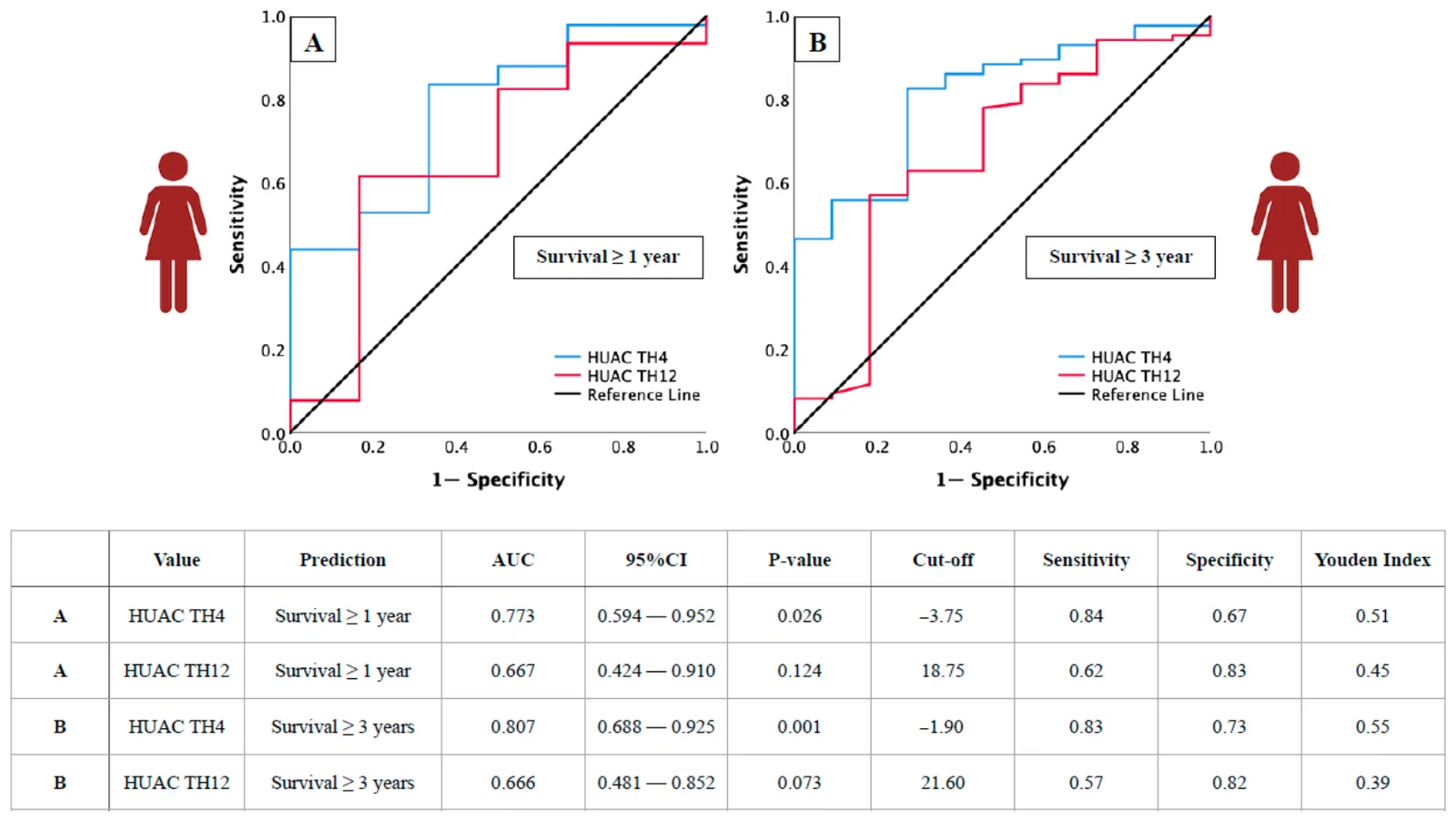
ROC Curves for HUAC at TH4 and TH12 in Female CABG Patients. Receiver-operating-characteristic (ROC) curves for HUAC measured at the fourth and twelfth thoracic vertebral levels in women. HUAC at TH4 showed higher AUC values for 1- and 3-year survival than TH12. Abbreviations: HUAC = Hounsfield Unit Average Calculation; TH = thoracic vertebra; AUC = area under the curve; ROC = receiver-operating-characteristic; CI = confidence interval.

**Figure 6:**
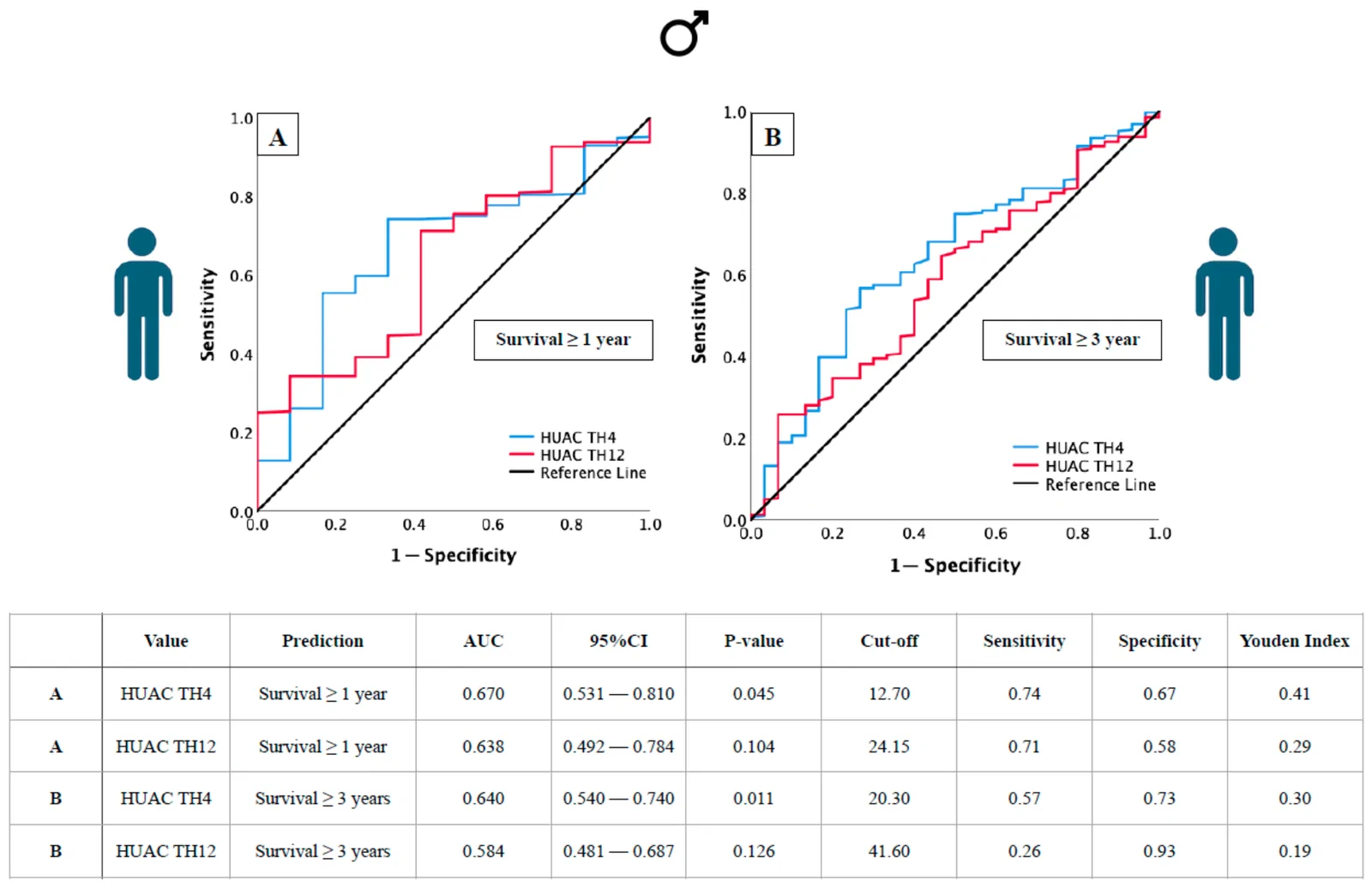
ROC Curves for HUAC at TH4 and TH12 in Male CABG Patients. Receiver-operating-characteristic (ROC) curves for HUAC measured at TH4 and TH12 in men. Predictive accuracy was lower than in women, with HUAC TH4 outperforming TH12 but with weaker overall discrimination. Abbreviations: HUAC = Hounsfield Unit Average Calculation; TH = thoracic vertebra; AUC = area under the curve; ROC = receiver-operating-characteristic; CI = confidence interval.

AUROC curves for HUAC measured at the fourth (TH4) and twelfth (TH12) thoracic levels are displayed in Figure 5 (women) and Figure 6 (men).

Across both sexes, HUAC at TH4 showed higher AUC values than HUAC at TH12. In women, HUAC at TH4 yielded an AUC of 0.773 (95% CI 0.594–0.952; *p* = 0.026) for 1-year survival. The cohort-derived threshold maximizing the Youden Index was −3.75 HU, with a sensitivity of 0.84, specificity of 0.67, and YI of 0.51. For 3-year survival, the AUC was 0.807 (95% CI 0.688–0.925; *p* = 0.001), with a cohort-derived threshold of −1.90 HU (sensitivity 0.83, specificity 0.73, YI 0.55). HUAC at TH12 yielded AUCs of 0.667 and 0.666 (*p* = 0.124 and *p* = 0.073), with corresponding cohort-derived thresholds of 18.75 HU and 21.60 HU and YI values of 0.45 and 0.39.

In men, predictive accuracy was lower overall. HUAC TH4 reached an AUC of 0.670 (95% CI 0.531–0.810; *p* = 0.045) for survival ≥ 1 year with a cohort-derived threshold of 12.70 HU (sensitivity 0.74, specificity 0.67, YI 0.41). For survival ≥ 3 years, the AUC was 0.640 (95% CI 0.540–0.740; *p* = 0.011) with a cut-off of 20.30 HU (sensitivity 0.57, specificity 0.73, YI 0.30). HUAC TH12 values were lower and mostly non-significant (AUC 0.638 and 0.584; *p* = 0.104 and 0.126), with corresponding cohort-derived thresholds of 24.15 HU and 41.60 HU and smaller YI (0.29 and 0.19).

### 4.7. Cox Regression Analysis and Sex Interaction Analysis

**Figure 7:**
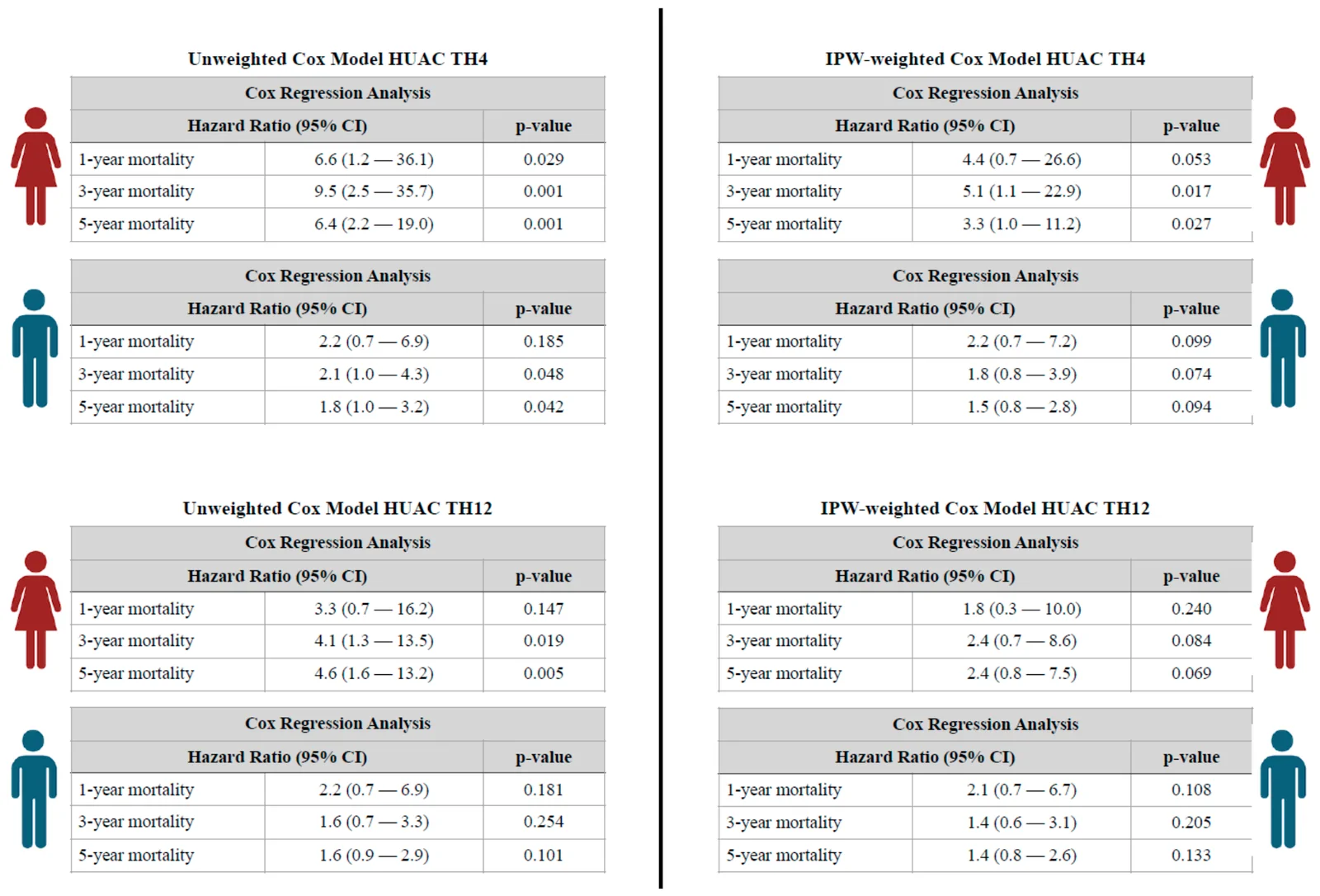
Sex-Specific Cox Regression Models for Mid- and Long-Term Mortality After CABG. Unweighted and inverse-probability-weighted (IPW) Cox regression models for 1-, 3-, and 5-year all-cause mortality according to HUAC at TH4 and TH12. Hazard ratios (HR) and 95% confidence intervals (CI) are shown for the lower HUAC quartile versus all others. Abbreviations: HUAC = Hounsfield Unit Average Calculation; TH = thoracic vertebra; CABG = coronary artery bypass grafting; IPW = inverse probability weighting; HR = hazard ratio; CI = confidence interval.

To confirm and quantify the prognostic impact of thoracic muscle density, sex-specific unweighted and IPW-weighted Cox regression analyses were performed for HUAC measured at both the TH4 and TH12 levels (Figure 7).

In women, low HUAC at TH4 was associated with higher mortality across all follow-up intervals. In the unweighted model, patients in the lower HUAC quartile had a 6.6-fold higher risk of 1-year mortality (95% CI 1.2–36.1; *p* = 0.029), a 9.5-fold higher risk at 3 years (95% CI 2.5–35.7; *p* = 0.001), and a 6.4-fold higher risk at 5 years (95% CI 2.2–19.0; *p* = 0.001) compared with women in higher HUAC quartiles. After IPW adjustment, the association remained statistically significant at 3- and 5-year follow-up. At the TH12 level, the predictive value was weaker: unweighted hazard ratios ranged from 2.2 to 1.8, reaching statistical significance only at longer follow-up (3 years *p* = 0.048; 5 years *p* = 0.042). After IPW adjustment, the association attenuated and lost significance.

In men, HUAC also correlated with mortality risk, but the magnitude was considerably smaller. At TH4, unweighted hazard ratios for the lower quartile were approximately 2.0–2.5 across the three follow-up periods, and most models failed to reach statistical significance after IPW correction. At TH12, no consistent relationship was observed.

IPW substantially improved covariate balance across the sex-specific models (Supplementary Table S1). In men, all post-weighting absolute SMDs were <0.10 for both TH4 and TH12. In women, balance was likewise markedly improved, although minor residual imbalance remained for creatinine and EuroSCORE II at TH4 (SMD 0.144 and 0.111, respectively) and for creatinine at TH12 (SMD 0.105). All remaining post-weighting SMDs were <0.10.

Formal interaction analyses (Supplementary Table S2) supported a sex-dependent association between low HUAC at TH4 and mortality. The sex × low-HUAC interaction was not significant at 1 year (interaction HR 0.33, 95% CI 0.04–2.57; *p* = 0.291), but reached statistical significance at 3 years (interaction HR 0.21, 95% CI 0.05–0.97; *p* = 0.046) and 5 years (interaction HR 0.28, 95% CI 0.08–0.97; *p* = 0.045). With women as the reference category, interaction HRs below 1 indicate a weaker association between low HUAC and mortality in men than in women. In contrast, no significant sex × low-HUAC interaction was observed for TH12 at 1, 3, or 5 years (all *p* > 0.05).

### 4.8. Incremental Prognostic Value Beyond EuroSCORE II

To determine whether HUAC provided prognostic information beyond established surgical risk assessment, models incorporating low HUAC were compared with EuroSCORE II alone (Supplementary Table S3). In the overall cohort, addition of low TH4 HUAC consistently improved model discrimination. The C-statistic increased from 0.748 to 0.788 at 1 year (ΔC = +0.039; LR *p* = 0.014), from 0.665 to 0.711 at 3 years (ΔC = +0.046; LR *p* < 0.001), and from 0.658 to 0.690 at 5 years (ΔC = +0.032; LR *p* < 0.001).

The incremental gain associated with TH4 was most pronounced in women. Addition of low TH4 HUAC increased the C-statistic from 0.722 to 0.783 at 1 year (ΔC = +0.061; LR *p* = 0.022), from 0.679 to 0.795 at 3 years (ΔC = +0.115; LR *p* < 0.001), and from 0.707 to 0.788 at 5 years (ΔC = +0.081; LR *p* < 0.001). In men, improvements in discrimination were smaller, with ΔC values of +0.050, +0.038, and +0.024 at 1, 3, and 5 years, respectively.

In contrast, addition of low TH12 HUAC did not consistently improve discrimination in the overall cohort, despite improvement in model fit at 5 years. Sex-stratified analyses suggested modest incremental discrimination for TH12 in women at longer follow-up, whereas no improvement in C-statistic was observed in men.

## 5. Discussion

This study demonstrates that thoracic skeletal muscle attenuation (HUAC), measured on routine preoperative CT scans, provides prognostic information for postoperative and long-term survival in patients undergoing CABG. Among the two levels analyzed, HUAC at TH4 consistently showed superior discriminative ability compared with TH12. Importantly, low TH4 HUAC provided incremental prognostic information beyond EuroSCORE II, with the largest improvement in model discrimination observed in women. Together with the formal sex-by-HUAC interaction at 3- and 5-year follow-up, these findings support a sex-dependent prognostic relevance of upper thoracic muscle quality.

### 5.1. Clinical Relevance of HUAC in Cardiac Surgery

Skeletal muscle represents far more than a structural tissue: it is a metabolic and immunologic organ that influences systemic homeostasis, energy balance, and inflammation [23]. Surgical stress, ischemia–reperfusion injury, and the inflammatory cascade of cardiopulmonary bypass all impose a metabolic burden that demands substantial reserve capacity [24].

In contrast to traditional anthropometric surrogates of frailty such as BMI, grip strength, or gait speed, HUAC quantifies muscle quality directly [9]. It reflects the degree of intramuscular fat infiltration, a hallmark of chronic catabolic states, mitochondrial dysfunction, and impaired protein turnover [25]. Fatty infiltration of muscle alters contractile efficiency and impairs glucose and lipid metabolism, thereby exacerbating insulin resistance and systemic inflammation [26].

Previous cardiac surgery studies have mostly examined muscle cross-sectional area [8] rather than attenuation [27]. However, area-based indices measure muscle quantity but ignore its composition. The present study represents a secondary analysis of the same cohort of 479 patients previously reported in our study of thoracic HUAC after CABG [27]. The previous analysis focused exclusively on HUAC measured at TH12 and its association with postoperative morbidity and mortality. In contrast, the present study additionally evaluates TH4, directly compares the prognostic relevance of TH4 and TH12, formally assesses sex × low-HUAC interactions, and examines the incremental prognostic value of low HUAC beyond EuroSCORE II. Thus, although the underlying patient cohort and selected clinical and outcome data are shared, the present study addresses a distinct research question focusing on anatomical level specificity and sex-dependent prognostic associations. Our findings support the growing evidence that muscle attenuation may be more prognostically informative than muscle area because it captures qualitative aspects of muscle composition in addition to muscle quantity. This is consistent with reports in oncology [28,29] and kidney transplantation [30], where low HUAC predicted postoperative mortality and complications. Importantly, low TH4 HUAC also provided incremental prognostic information beyond EuroSCORE II, particularly in women, supporting its potential role as a complementary marker of physiological vulnerability.

The strength of HUAC lies in its clinical feasibility. When preoperative CT imaging is clinically available, HUAC can be extracted from existing scans without additional imaging or radiation exposure. The measurement is rapid, reproducible, and easily integrated into image-processing pipelines.

In practical terms, identifying patients with low HUAC could prompt targeted prehabilitation, protein supplementation, and respiratory training, or trigger early postoperative mobilization and nutritional support [31]—all interventions known to mitigate frailty-related risk.

### 5.2. Why HUAC at TH4 Outperformed HUAC at TH12

The superior discriminative performance of HUAC measured at TH4 compared with TH12 likely reflects fundamental differences in both anatomical representation and physiological sensitivity.

The TH4 level represents the upper thoracic paraspinal musculature, which may differ from lower thoracic muscle in its composition and susceptibility to systemic metabolic decline and chronic cardiopulmonary stress [32].

By contrast, HUAC measured at TH12 reflects lower thoracic and lumbar musculature—muscles more influenced by spinal stability, local fat deposition, and positional variation. The lower thoracic region is anatomically adjacent to the diaphragm, liver, and visceral fat compartments; this proximity introduces measurement noise and potential partial-volume effects from surrounding adipose tissue. In older or obese patients, the signal-to-noise ratio at TH12 may therefore be inferior, blunting its predictive accuracy [33].

From a physiological standpoint, differences in muscle attenuation between thoracic levels may reflect regional differences in muscle composition and vulnerability to systemic disease. Although respiratory and postural muscle integrity is relevant to postoperative recovery after cardiac surgery [34,35], these muscle groups were not directly assessed in the present study. As our measurements were confined to the paraspinal muscles, no conclusions regarding respiratory muscle quality or function can be drawn from our data.

Our results align with observations from non-cardiac cohorts showing that muscle attenuation at higher thoracic levels more accurately reflects all-cause mortality than lumbar indices. In lung cancer [36] and chronic obstructive pulmonary disease [37] cohorts, HUAC near the pectoralis or paraspinal muscles predicted survival independently of BMI or muscle area. Together, these data suggest that HUAC at TH4 may represent an imaging-derived marker of systemic vulnerability, although the biological basis for its stronger prognostic association requires further investigation.

### 5.3. Why HUAC Predicted Outcomes More Strongly in Women

The finding that HUAC carried a stronger prognostic signal in women than in men was further supported by formal interaction testing. Specifically, a significant sex × low-HUAC interaction was observed for TH4 at 3- and 5-year follow-up, whereas no significant interaction was observed at TH12. These findings provide statistical evidence that the prognostic association of low TH4 muscle attenuation differs by sex, with a stronger association in women.

Several biological and methodological factors may potentially contribute to this difference.

Women generally possess lower absolute muscle mass but exhibit greater intrafascial fat deposition within skeletal muscle [38]. Accordingly, a decline in HUAC could represent a proportionally greater loss of functional reserve in women, potentially contributing to its stronger prognostic association. Even modest reductions in muscle density might therefore reflect clinically relevant vulnerability in this population.

Furthermore, hormonal and metabolic differences may provide a potential biological explanation. After menopause, estrogen deficiency accelerates myosteatosis through alterations in lipid oxidation, mitochondrial function, and inflammatory signaling. Estrogen also modulates capillary perfusion and glucose uptake in muscle tissue [39]. These mechanisms provide a biologically plausible framework through which age-related hormonal changes could influence muscle attenuation and its prognostic relevance in women. However, menopausal status and circulating hormone levels were not available in the present cohort; therefore, these mechanisms remain speculative and cannot be directly inferred from our data.

Additionally, the male thoracic musculature is more voluminous and may require a greater absolute reduction in density before functional consequences emerge [40]. Differences in muscle mass and composition could therefore contribute to the lower discriminatory capacity of HUAC observed in men. Conversely, thoracic muscle attenuation may more closely reflect physiological vulnerability in women, although this interpretation requires confirmation in independent cohorts.

The clinical implications may be relevant. Female patients undergoing CABG typically present older, with higher EuroSCORE II, smaller coronary vessels, and higher rates of postoperative complications—an established “female disadvantage” in cardiac surgery [41]. Importantly, emerging evidence suggests that the interaction between sex and aging may extend beyond systemic and muscular phenotypes to vascular disease characteristics. Recent histopathological data indicate that sex and age may modify the association between dyslipidemic profiles and carotid plaque instability, with women exhibiting greater susceptibility to plaque instability in the presence of specific atherogenic lipid profiles [42]. These observations further emphasize that cardiovascular vulnerability may reflect complex interactions between sex, aging, metabolic status, and vascular phenotype. In this context, HUAC may provide additional information on the physiological component of this disparity, offering a quantifiable marker of biological age that goes beyond chronological or anatomical metrics.

From a translational perspective, integrating HUAC into preoperative evaluation may eventually contribute to sex-tailored risk stratification. In women with low HUAC, structured prehabilitation, resistance training (when possible), or nutritional interventions could represent potential strategies to address impaired muscle quality. However, whether HUAC-guided interventions improve postoperative outcomes cannot be determined from the present observational study and requires prospective evaluation.

## 6. Limitations

This study has several limitations.

First, its retrospective single-center design entails potential selection bias and limits generalizability. Although IPW substantially improved the balance of measured covariates, minor residual imbalance remained in some of the female models, and residual or unmeasured confounding cannot be excluded. In particular, detailed measures of nutritional status, comprehensive body composition and functional frailty, heart failure severity, and metabolic disease burden were not incorporated into the weighting model. Accordingly, the weighted analyses should be interpreted as adjusted observational associations rather than evidence of causality.

Second, HUAC was assessed at two discrete thoracic levels (TH4 and TH12), rather than through full-volume segmentation, which may not fully capture regional variability in muscle composition. The definition of low HUAC as the lowest sex-specific quartile was cohort-derived and therefore identifies approximately 25% of each sex by design. Similarly, the thresholds obtained from the ROC analyses were derived and evaluated within the same cohort and may therefore yield optimistic estimates of discrimination. Neither the quartile-based definition nor the ROC-derived thresholds should be interpreted as diagnostic or clinically established cut-offs. External validation in independent CABG cohorts is required before their application to individual risk stratification.

Third, technical factors such as scanner type, reconstruction kernel, and contrast phase may influence attenuation values, although standardized institutional protocols minimized this variability.

Fourth, the female subgroup was relatively small (n = 97), with a limited number of mortality events, reflecting the typical sex distribution in CABG cohorts. Consequently, sex-specific analyses, particularly the effect estimates in women with their relatively wide confidence intervals, should be considered exploratory. Although formal interaction testing supported sex-dependent differences in the prognostic association of low HUAC at TH4 at 3- and 5-year follow-up, these findings require external validation in larger, independent cohorts.

Finally, this study demonstrates association rather than causation. Low HUAC likely reflects a broader systemic vulnerability—encompassing inflammation, malnutrition, or hormonal factors—rather than a direct causal mechanism. Prospective validation and interventional studies are needed to determine whether improving muscle quality can modify surgical risk.

## 7. Conclusions

Low thoracic skeletal muscle attenuation, particularly at TH4, is associated with mid- and long-term mortality after CABG and provides prognostic information beyond EuroSCORE II. Its prognostic relevance was particularly pronounced in women, supported by formal sex-by-HUAC interaction testing and greater incremental improvement in model discrimination. As an objective imaging-derived marker available from routine preoperative CT, HUAC may complement established surgical risk assessment by capturing aspects of physiological reserve not represented by conventional risk scores. External validation is required before integration into clinical risk-prediction models.

## Figures and Tables

**Table 1:** Sex-Specific HUAC Quartile Thresholds at TH4 and TH12. Sex-specific HUAC quartile thresholds used to define low muscle attenuation. Patients in the lowest sex-specific quartile (Q1) were classified as having low muscle attenuation. Abbreviations: HU = Hounsfield units; HUAC = Hounsfield Unit Average Calculation; TH = thoracic vertebra.

	HUAC TH4	HUAC TH12	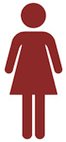
Q1	<−1.50	<11.80	
Q2	−1.50–11.89	11.80–22.29	
Q3	11.90–20.75	22.30–34.40	
Q4	>20.75	>34.40	
	HUAC TH4	HUAC TH12	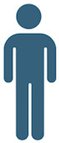
Q1	<11.50	<21.80	
Q2	11.50–21.75	21.80–32.50	
Q3	21.76–30.33	32.51–41.40	
Q4	>30.33	>41.40	

**Table 2:** Baseline Clinical Characteristics. Baseline demographics, anthropometric data and comorbidities for the study population, stratified by sex. Continuous variables are presented as mean ± SD or median [IQR]; categorical variables as n (%). Abbreviations: BMI = body-mass index; BSA = body-surface area; COPD = chronic obstructive pulmonary disease; EF = ejection fraction; NYHA = New York Heart Association; EuroSCORE II = European System for Cardiac Operative Risk Evaluation II.

	Total	Men	Women	*p*-Value
No. (%)				
Total	479 (100.0)	382 (100.0)	97 (100.0)	-
Age > 70 years	241 (50.3)	190 (49.7)	51 (52.6)	0.617
BMI ≥ 25.0 kg/m^2^	354 (73.9)	287 (75.1)	67 (69.1)	0.225
Arterial Hypertension	432 (90.2)	346 (90.6)	86 (88.7)	0.571
Diabetes Mellitus	165 (34.4)	130 (34.0)	35 (36.1)	0.704
Dyslipidaemia	423 (88.3)	335 (87.7)	88 (90.7)	0.408
Familiar History of CV Disease	123 (25.7)	92 (24.1)	31 (32.0)	0.113
PAOD	59 (12.3)	47 (12.3)	12 (12.4)	0.986
COPD				
I°	51 (10.6)	41 (10.7)	10 (10.3)	0.915
II°	16 (3.3)	14 (3.7)	2 (2.1)	
III°	5 (1.0)	4 (1.0)	1 (1.0)	
AF	41 (8.6)	33 (8.6)	8 (8.2)	0.902
Active Smoking	74 (15.4)	63 (16.5)	11 (11.3)	0.210
Active Cancer	32 (6.7)	28 (7.3)	4 (4.1)	0.259
Prior Stroke	43 (9.0)	36 (9.4)	7 (7.2)	0.497
Prior Heart Failure	64 (13.4)	56 (14.7)	8 (8.2)	0.097
Angina Pectoris	331 (69.1)	257 (67.3)	74 (76.3)	0.086
NYHA				
I	138 (28.8)	118 (30.9)	20 (20.6)	0.004
II	183 (38.2)	151 (39.5)	32 (33.0)	
III	90 (18.8)	60 (15.7)	30 (30.9)	
IV	68 (14.2)	53 (13.9)	15 (15.5)	
LVEF				
≥55%	379 (79.1)	293 (76.7)	86 (88.7)	0.069
41–54%	57 (11.9)	52 (13.6)	5 (5.2)	
31–40%	22 (4.6)	19 (5.0)	3 (3.1)	
≤30%	21 (4.4)	18 (4.7)	3 (3.1)	
Mitral Valve Regurgitation				
No	158 (33.0)	127 (33.2)	31 (32.0)	0.404
I°	269 (56.2)	217 (56.8)	52 (53.6)	
II°	51 (10.6)	38 (10.0)	14 (14.4)	
III°	0 (0.0)	0 (0.0)	0 (0.0)	
Tricuspid Valve Regurgitation				
No	247 (51.6)	200 (52.4)	47 (48.5)	0.855
I°	218 (45.5)	171 (44.8)	47 (48.5)	
II°	13 (2.7)	10 (2.6)	3 (3.1)	
III°	1 (0.2)	1 (0.3)	0 (0.0)	
Left Main Lesion ≥ 50%	199 (41.5)	170 (44.5)	29 (29.9)	0.009
Median [IQR]				
Age (years)	70.0 [14.0]	69.0 [15.0]	70.0 [12.8]	0.068
Height (cm)	172.0 [11.0]	175.0 [9.0]	161.0 [9.0]	<0.001
Weight	82.0 [18.0]	84.0 [17.0]	70.0 [16.8]	<0.001
BMI (kg/m^2^)	27.4 [5.5]	27.6 [5.5]	26.9 [5.8]	0.265
BSA (m^2^)	2.0 [0.3]	2.0 [0.2]	1.8 [0.2]	<0.001
EuroSCORE II	1.6 [1.9]	1.5 [1.8]	2.2 [2.5]	<0.001
Creatinine (mg/dL)	1.0 [0.3]	1.0 [0.3]	0.8 [0.3]	<0.001
LDH (U/L)	204.0 [50.0]	203.0 [48.0]	207.5 [51.8]	0.262
CRP (mg/dL)	0.2 [0.5]	0.2 [0.5]	0.2 [0.5]	0.592
Hemoglobin (g/dL)	14.2 [2.1]	14.4 [2.0]	13.4 [1.2]	<0.001

**Table 3:** Intraoperative Characteristics. Intraoperative parameters including graft configuration, operative times, and transfusion requirements, stratified by sex. Abbreviations: CABG = coronary artery bypass grafting; LIMA = left internal mammary artery; RIMA = right internal mammary artery; BIMA = bilateral internal mammary artery; IABP = intra-aortic balloon pump; CPB = cardiopulmonary bypass; RBC = red blood cell; FFP = fresh frozen plasma.

	Total	Men	Women	*p*-Value
No. (%)				
On-pump CABG	439 (91.6)	353 (92.4)	86 (88.7)	0.233
CABG via Left Thoracotomy	7 (1.5)	5 (1.3)	2 (2.1)	0.581
ITA Configuration				
None	12 (2.5)	7 (1.8)	5 (5.2)	0.085
LIMA	300 (62.6)	235 (61.5)	65 (67.0)	
RIMA	15 (3.1)	11 (2.9)	4 (4.1)	
BIMA	152 (31.7)	129 (33.8)	23 (23.7)	
IABP	4 (0.8)	3 (0.8)	1 (1.0)	0.812
Mean ± SD				
Red Blood Cell Concentrate	0.3 ± 0.9	0.1 ± 0.5	0.8 ± 1.6	<0.001
Platelet Concentrate	0.0 ± 0.2	0.0 ± 0.2	0.0 ± 0.3	0.982
Fresh Frozen Plasma	0.1 ± 0.8	0.1 ± 0.6	0.2 ± 1.3	0.445
Median [IQR]				
Perfusion Time (min)	93.0 [49.0]	93.5 [48.5]	92.0 [49.0]	0.701
Clamping Time (min)	49.0 [27.0]	48.5 [26.3	51.0 [32.5]	0.659
Arterial Grafts	2.0 [1.0]	2.0 [1.0]	1.0 [1.0]	0.492
Venous Grafts	1.0 [2.0]	1.0 [2.0]	1.0 [2.0]	0.717

**Table 4:** Postoperative Outcomes. Postoperative complications, 30-day mortality, and hospital course. Values are presented as n (%) or mean ± SD. Abbreviations: MI = myocardial infarction; AF = atrial fibrillation; AKI = acute kidney injury; ECLS = extracorporeal life support; ICU = intensive care unit; LCOS= Low cardiac output syndrome.

	Total	Men	Women	*p*-Value
No. (%)				
30-day mortality	9 (1.9)	7 (1.8)	2 (2.1)	0.882
LCOS requiring ECMO	6 (1.3)	5 (1.3)	1 (1.0)	0.826
Myocardial Infarction	8 (1.7)	5 (1.3)	3 (3.1)	0.221
AKI requiring Haemodialysis	7 (1.5)	6 (1.6)	1 (1.0)	0.692
Sternal Wound Infection	17 (3.5)	13 (3.4)	4 (4.1)	0.732
AF (new onset)	110 (23.0)	89 (23.3)	21 (21.6)	0.730
Reexploration for Bleeding	8 (1.7)	8 (2.1)	0 (0.0)	0.151
Prolonged Ventilation ≥ 48 h	26 (5.4)	20 (5.2)	6 (6.2)	0.712
Reintubation rate	17 (3.5)	12 (3.1)	5 (5.2)	0.339
Disabling Stroke	8 (1.7)	5 (1.3)	3 (3.1)	0.221
Graft Occlusion	7 (1.5)	4 (1.0)	3 (3.1)	0.134
Mean ± SD				
Invasive Ventilation Time (h)	13.2 ± 45.4	13.5 ± 49.6	12.2 ± 21.5	0.800
ICU Stay (d)	3.3 ± 5.1	3.4 ± 5.6	3.1 ± 2.9	0.683

## Data Availability

The data presented in this study are available from the corresponding author upon reasonable request. Public disclosure of the underlying patient data is restricted by the ethics committee approval for ethical and privacy reasons.

## Supplementary Materials

The following supporting information can be downloaded at https://www.mdpi.com/article/10.3390/medsci14050569/s1, Supplementary Table S1: Covariate Balance Before and After Inverse Probability Weighting; Supplementary Table S2: Formal Sex-by-HUAC Interaction Analysis for 1-, 3-, and 5-Year Mortality After CABG; Supplementary Table S3: Incremental Prognostic Value of Low HUAC Beyond EuroSCORE II.
