# Supplementary material for: Thoracic Skeletal Muscle Attenuation Predicts Sex-Specific Mortality After Coronary Artery Bypass Grafting—Sex-Specific Insights into Risk Stratification and Frailty Assessment
Source: Med Sci (Basel). 2026 Sep 14;14(5):569. doi: 10.3390/medsci14050569 (PMC13609366; doi:10.3390/medsci14050569)
Supplement: Supplementary file 1 [file medsci-14-00569-s001.zip › Supplementary Table S1.pdf]

Supplementary Table S1. Covariate Balance Before and After Inverse Probability Weighting

HUAC level	Sex	Covariate	SMD before IPW	SMD after IPW
TH4	Women	Age	0.878	0.040
TH4	Women	Creatinine	0.462	0.144
TH4	Women	Hemoglobin	0.043	0.049
TH4	Women	EuroSCORE II	0.034	0.111
TH4	Men	Age	0.453	0.014
TH4	Men	Creatinine	0.189	0.050
TH4	Men	Hemoglobin	0.219	0.011
TH4	Men	EuroSCORE II	0.058	0.049
TH12	Women	Age	0.585	0.054
TH12	Women	Creatinine	0.427	0.105
TH12	Women	Hemoglobin	0.101	0.097
TH12	Women	EuroSCORE II	0.200	0.095
TH12	Men	Age	0.483	0.075
TH12	Men	Creatinine	0.143	0.013
TH12	Men	Hemoglobin	0.150	0.029
TH12	Men	EuroSCORE II	0.121	0.002

Absolute standardized mean differences (SMDs) for covariates included in the propensity-score models, shown before and after inverse probability weighting (IPW), separately by thoracic level and sex. Absolute SMD <0.10 was considered indicative of adequate covariate balance. Weighting substantially improved covariate balance. All post-weighting SMDs were <0.10 in men. In women, minor residual imbalance remained for creatinine and EuroSCORE II at TH4 (SMD 0.144 and 0.111, respectively) and for creatinine at TH12 (SMD 0.105).

Abbreviations: HUAC = Hounsfield Unit Average Calculation; TH = thoracic vertebral level; IPW = inverse probability weighting; SMD = standardized mean difference.
