# Supplementary material for: Thoracic Skeletal Muscle Attenuation Predicts Sex-Specific Mortality After Coronary Artery Bypass Grafting—Sex-Specific Insights into Risk Stratification and Frailty Assessment
Source: Med Sci (Basel). 2026 Sep 14;14(5):569. doi: 10.3390/medsci14050569 (PMC13609366; doi:10.3390/medsci14050569)
Supplement: Supplementary file 1 [file medsci-14-00569-s001.zip › Supplementary Table S2.pdf]

Supplementary Table S2. Formal Sex-by-HUAC Interaction Analysis for 1-, 3-, and 5-Year Mortality After CABG

Thoracic level	Follow-up	Interaction HR	95% CI	p for interaction
TH4	1 year	0.33	0.04–2.57	0.291
TH4	3 years	0.21	0.05–0.97	0.046
TH4	5 years	0.28	0.08–0.97	0.045
TH12	1 year	0.67	0.09–4.83	0.695
TH12	3 years	0.37	0.09–1.50	0.163
TH12	5 years	0.35	0.10–1.17	0.088

Cox proportional hazards models included low HUAC (lowest sex-specific quartile), sex, and the sex-by-low-HUAC interaction term. Women served as the reference category for sex. The interaction HR represents the multiplicative sex-by-low-HUAC interaction term (HR for the low-HUAC association in men relative to the corresponding association in women). Thus, an interaction HR <1 indicates a weaker association between low HUAC and mortality in men than in women.

Abbreviations: CABG = coronary artery bypass grafting; HUAC = Hounsfield Unit Average Calculation; TH = thoracic vertebral level; HR = hazard ratio; CI = confidence interval.
