# Supplementary material for: Thoracic Skeletal Muscle Attenuation Predicts Sex-Specific Mortality After Coronary Artery Bypass Grafting—Sex-Specific Insights into Risk Stratification and Frailty Assessment
Source: Med Sci (Basel). 2026 Sep 14;14(5):569. doi: 10.3390/medsci14050569 (PMC13609366; doi:10.3390/medsci14050569)
Supplement: Supplementary file 1 [file medsci-14-00569-s001.zip › Supplementary Table S3.pdf]

Supplementary Table S3. Incremental Prognostic Value of Low HUAC Beyond EuroSCORE II

Population	Follow-up	HUAC level	n	Deaths	C-statistic EuroSCORE II	C-statistic + low HUAC	ΔC	LR χ²	p value
All	1 year	TH4	479	18	0.748	0.788	+0.039	6.08	0.014
All	1 year	TH12	479	18		0.730	-0.018	2.47	0.116
All	3 years	TH4	479	41	0.665	0.711	+0.046	12.96	<0.001
All	3 years	TH12	479	41		0.652	-0.013	3.61	0.057
All	5 years	TH4	479	64	0.658	0.690	+0.032	12.32	<0.001
All	5 years	TH12	479	64		0.652	-0.006	6.31	0.012
Women	1 year	TH4	97	6	0.722	0.783	+0.061	5.23	0.022
Women	1 year	TH12	97	6		0.737	+0.015	1.89	0.169
Women	3 years	TH4	97	11	0.679	0.795	+0.115	13.14	<0.001
Women	3 years	TH12	97	11		0.731	+0.052	5.12	0.024
Women	5 years	TH4	97	14	0.707	0.788	+0.081	13.44	<0.001
Women	5 years	TH12	97	14		0.745	+0.038	6.57	0.010
Men	1 year	TH4	382	12	0.746	0.795	+0.050	1.62	0.204
Men	1 year	TH12	382	12		0.743	-0.003	1.22	0.270
Men	3 years	TH4	382	30	0.649	0.687	+0.038	3.54	0.060
Men	3 years	TH12	382	30		0.646	-0.004	1.01	0.314
Men	5 years	TH4	382	50	0.642	0.666	+0.024	3.89	0.049
Men	5 years	TH12	382	50		0.639	-0.004	2.44	0.118

Comparison of Cox proportional hazards models including EuroSCORE II alone versus EuroSCORE II plus sex-specific low HUAC (lowest quartile), separately for TH4 and TH12 and for 1-, 3-, and 5-year all-cause mortality. C-statistics represent Harrell's concordance index from horizon-specific Cox models with censoring at 1, 3, or 5 years. The likelihood-ratio (LR) test compares the nested EuroSCORE II model with the corresponding EuroSCORE II + low-HUAC model. Positive ΔC indicates improved discrimination after addition of low HUAC.

Abbreviations: HUAC, Hounsfield Unit Average Calculation; TH, thoracic vertebral level; LR, likelihood ratio.
